# Volume expansion mitigates Shiga toxin-producing *E. coli*-hemolytic uremic syndrome in children

**DOI:** 10.1007/s00467-023-06276-3

**Published:** 2024-01-19

**Authors:** Johannes Böckenhauer, Raphael Schild, Markus J. Kemper, Thomas Henne, Marie V. Stein, Jun Oh, Sebastian Loos

**Affiliations:** 1 University Medical Center Hamburg-Eppendorf, University Children’s Hospital, Martinistrasse 52, 20246 Hamburg, Germany; 2 Department of Pediatrics, Asklepios Klink Nord, Hamburg, Germany

**Keywords:** HUS, EHEC, STEC, Volume expansion, Hyperhydration, Outcome

## Abstract

**Background:**

Shiga toxin-producing *E. coli*-hemolytic uremic syndrome (STEC-HUS) is associated with high morbidity and relevant mortality. Previous small studies showed that volume expansion could improve the course and outcome of STEC-HUS. The aim of this single-center study was to evaluate the effect of volume expansion on the clinical course and outcome in STEC-HUS.

**Methods:**

Data of pediatric patients with STEC-HUS were analyzed retrospectively. Course and outcome of patients treated with volume expansion (VE) from 2019 to 2022 (*n* = 38) were compared to historical controls (HC) from 2009 to 2018 (*n* = 111).

**Results:**

Patients in the VE group had a significant relative median weight gain compared to HC (7.8% (3.4–11.3) vs. 1.2% (− 0.7–3.9), *p* < 0.0001) 48 h after admission. The need for dialysis was not reduced by VE (VE 21/38 (55.3%) vs. HC 64/111 (57.7%), *p* = 0.8). However, central nervous system involvement (impairment of consciousness, seizures, focal neurological deficits, and/or visual disturbances) was significantly reduced (VE 6/38 (15.8%) vs. HC 38/111 (34.2%), *p* = 0.039). None of the patients in the VE group died or developed chronic kidney disease (CKD) stage 5, whereas in the HC group, three patients died and three patients had CKD stage 5 at discharge.

**Conclusions:**

This study suggests that volume expansion may be associated with the mitigation of the acute course of STEC-HUS, especially severe neurological involvement and the development of CKD. Prospective trials should lead to standardized protocols for volume expansion in children with STEC-HUS.

**Graphical abstract:**

**Figure:**
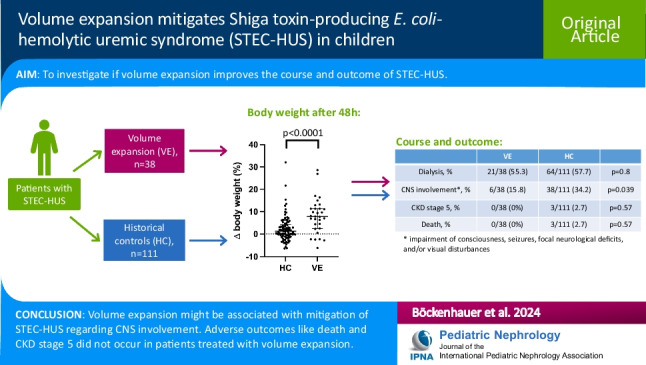
A higher resolution version of the Graphical abstract is available as Supplementary information

**Supplementary Information:**

The online version contains supplementary material available at 10.1007/s00467-023-06276-3.

## Introduction

Shiga toxin-producing *Escherichia coli* (STEC) can cause enterocolitis, and in about 15–20% of cases, the gastrointestinal phase is followed by hemolytic uremic syndrome (HUS) [1]. After STEC infection, Shiga toxin (Stx) crosses the intestinal barrier, enters the circulation, and induces multi-organ damage by activation of endothelial cells and induction of thrombo-inflammatory pathways [2]. The main target organs are the kidney and the central nervous system (CNS) leading to acute kidney injury (AKI) and severe neurological symptoms. STEC-HUS is clinically defined by non-immune hemolytic anemia, thrombocytopenia, and AKI [1]. Typically, children ≤ 5 years of age are affected by STEC-HUS. However, large numbers of adult cases can occur particularly in outbreak scenarios. STEC-HUS must be differentiated from complement-mediated/atypical HUS and HUS due to other triggers.

Treatment of STEC-HUS is focused on its complications since no causative treatment is available so far. The main therapeutical interventions are fluid and hypertension management, transfusions, kidney replacement therapy (KRT), and treatment of neurological symptoms like seizures. If the abovementioned therapies, including KRT, are available, the mortality of STEC-HUS is around 1–4% [3]. After the acute phase, patients are at risk of suffering from chronic kidney disease (CKD) stages 2–4 in around 10–20% of cases, CKD stage 5 in 3–4% of cases, and major neurological sequelae [3–6]. Even though major adverse outcomes like CKD stage 5 and death are rare events, improvement of the short- and long-term outcomes is needed.

At the beginning of HUS, the fluid status in most patients is characterized by dehydration after several days of prodromal gastroenteritis. On the other hand, by definition, these patients develop AKI with potential oligo-/anuria and are subsequentially at risk of fluid overload, if large fluid volumes are administered. Therefore, fluid management is often fluid restrictive or aiming for euvolemia in STEC-HUS. However, dehydration and hemoconcentration probably substantially add to the direct effect of Stx on organ damage and AKI via poor organ perfusion [7]. Several studies have shown that hemoconcentration in STEC infections is associated with a higher risk of development of STEC-HUS and severity of subsequent STEC-HUS [7–15].

Early fluid administration in STEC gastroenteritis leads to a reduction of STEC infections that progress to HUS and mitigates the severity of AKI [7, 11, 16, 17]. A large multicenter study with over 1000 patients with STEC infection will further assess these effects in a randomized trial with a target weight gain of 10% for the volume expansion group [18].

Based on the concept that volume expansion in patients with STEC-HUS corrects intravascular volume depletion and improves organ perfusion, Ardissino et al. showed in a cohort of 38 patients that volume expansion in established STEC-HUS significantly reduced the need for dialysis compared to historical controls (Table 2) [19]. In this study, STEC-HUS patients were treated with intravenous fluid to reach a target weight of working weight (known or estimated weight before HUS) + 10% within 48 h after admission. A significant reduction of dialysis treatment was also observed by Bonany et al. (Table 2) in patients (*n* = 16) treated with intravenous fluid 10–30 mL/kg body weight over 3 h on admission [20]. In both studies, CNS involvement was reduced and no deaths occurred. However, this was not statistically significant. So far, larger clinical trials are lacking, and no recommendations or guidelines with standardized protocols for volume expansion in STEC-HUS exist. The aim of this retrospective study was to analyze the course of disease and outcome of STEC-HUS patients treated with volume expansion at our center.

## Material and methods

### Cohort

The cohort comprises all pediatric patients treated for STEC-HUS at the University Medical Center Hamburg from January 2009 to 2022. Patients with complement/atypical HUS and/or HUS related to other infections were excluded. Medical records were analyzed retrospectively. At the beginning of 2019, a protocol for volume expansion (see below) was implemented. All STEC-HUS patients from 2019 to 2022 (all treated with volume expansion) were compared to historical controls from 2009 to 2018 (treated without target weight-based volume expansion) regarding the clinical course and outcome of HUS.

Stool was analyzed using enriched cultures to detect STEC and immunoassay or polymerase chain reaction (PCR) for the detection of Stx. Some patients in this study were already included in previous publications [15, 21]. Estimated glomerular filtration rate (eGFR) was used to assign patients to the appropriate CKD stage and was calculated using the appropriate Schwartz formula [22, 23]. CKD was categorized according to the National Kidney Foundation Kidney Disease Outcomes Quality Initiative (NKF-KDOQI) guidelines into stages 1–5 [24]. The study was approved by the federal state ethical committee (PV3975, WF-016/19).

### Clinical definitions

The following clinical definitions were applied: (i) for HUS, hemolytic anemia (minimum hemoglobin below the lower limit of the normal range), thrombocytopenia (minimum thrombocytes < 150 × 10^9^/L) or evidence of platelet consumption, and serum-creatinine above the upper limit of the age-dependent normal range; and (ii) for CNS involvement, major neurological symptoms like impairment of consciousness (stupor/ coma), seizures, focal neurological deficits, and/or visual disturbances (double/blurry vision).

### Treatment protocol for volume expansion

We modified the protocol from Ardissino et al. [19]. In the volume expansion group (VE), patients were treated with intravenous fluid (saline 0.9% or balanced solution) starting on admission to reach a target weight defined as baseline weight + 5% within 48 h. Baseline weight was the weight before illness, if known, or estimated by clinical grade of dehydration. Dehydration was assumed to be a maximum of 5% to limit the total maximum weight gain to 10% to avoid fluid overload.

### Statistics

Categorical variables (number and percentage) and continuous variables (median and interquartile range (IQR)) are presented. Fisher’s exact test was used to analyze categorical data. Mann–Whitney *U* test was used for comparison of continuous variables. A *p* value of < 0.05 was considered statistically significant. Data were analyzed using PRISM (Version 9, GraphPad, USA).

## Results

### Cohort

In total, 149 patients with STEC-HUS treated at our center from 2009 to 2022 were included in the study. The patients were 3.9 (1.7–8.1) years old. Seventy (47%) patients were male, and 79 (53%) patients were female. Analysis of the stool by culture and/or PCR/immunoassay was positive for STEC and/or Stx in 113 (76%) patients. CNS involvement was evident in 43 (29%) patients and KRT was performed in 85 (57%) patients for a median duration of 10 (7–15) days, in those without dialysis at discharge. Red blood cell transfusions were given to 121 (81%) of the patients. Three patients (2%) had CKD stage 5 and were on dialysis at discharge and mortality was 2%.

### Acute disease in the volume expansion group vs. historical controls

The group of historical controls (HC) consisted of 111 patients from 2009 to 2018 (11.1 cases/year) and the volume expansion group (VE) of 38 patients treated from 2019 to 2022 (9.5 cases/year). The baseline characteristics of both groups are presented in Table 1. Historical controls were significantly older and had higher hemoglobin on admission compared to the VE group (Table 1). Both differences were due to 33 patients from the *E. coli* O104 outbreak in 2011 (age 12.0 years (5.7–13.3)) in the HC group. When these patients were excluded, the HC group without O104 cases (*n* = 78) and the VE group did not differ regarding hemoglobin on admission (9.0 g/dL (7.1–10.3) vs. 8.3 g/dL (6.3–9.6), *p* = 0.15) and age (3.2 years (1.5–5.1) vs. 2.7 years (1.3–5.7), *p* = 0.43). When O104 cases were compared to the other patients in the HC group, they exhibited significantly higher hemoglobin on admission (10.8 g/dL (9.5–11.5) vs. 9.0 g/dL (7.1–10.3), *p* < 0.0001). However, the other parameters on admission (lactate dehydrogenase (LDH), creatinine, platelets) did not significantly differ (data not shown). Additionally, measures of the course (need for dialysis, duration of dialysis, and neurological involvement; data not shown) and outcome (CKD stage 5 or death: HC without O104 cases 5/78 (6.4%) vs. O104 1/33 (3%), *p* = 0.47) did not show any differences between O104 cases and the rest of the HC group. Therefore, O104 cases were included in the HC group in the following analysis.

**Table 1:** Demographic, laboratory (median, IQR), and clinical data of STEC-HUS patients according to the treatment group. To convert values for creatinine to µmol/L multiply by 88.4

		Volume expansion (*n* = 38)	Historical controls (*n* = 111)	VE vs. HC
	Age, years	2.7 (1.3–5.7)	4.1 (1.9–10.2)	*p* = 0.0072
	Males, *n* (%)	18 (47)	52 (47)	*p* = 0.96
Admission	Hemoglobin, g/dL	8.3 (6.3–9.6)	9.5 (8.2–10.9)	*p* = 0.0046
	LDH, U/L	1928 (1325–2784)	2129 (1425–2753)	*p* = 0.59
	Creatinine, mg/dL	2.5 (0.9–4.1)	3.0 (1.3–5.0)	*p* = 0.18
	Thrombocytes, × 10^9^/L	48.0 (25.3–94.5)	48.0 (31.0–74.5)	*p* = 0.89
Minimum/maximum during course of disease	Hemoglobin, g/dL	6.2 (5.9–6.8)	6.2 (5.6–6.7)	*p* = 0.38
	LDH, U/L	2167 (1430–3119)	2537 (1920–3178)	*p* = 0.15
	Creatinine, mg/dL	3.8 (1.3–6.2)	4.9 (2.4–7.9)	*p* = 0.13
	Thrombocytes, × 10^9^/L	40.5 (20.3–83.8)	31.0 (19.5–45.5)	*p* = 0.064
Clinical course	Δ body weight 48 h, %	7.8 (3.4–11.3)^a^	1.2 (–0.7–3.9)^b^	*p* < 0.0001
	Dialysis, *n* (%)	21 (55.3)	64 (57.7)	*p* = 0.8
	Duration of dialysis, days	11 (5–16)^c^	10 (7–15)^d^	*p* = 0.86
	CNS involvement, *n* (%)	6 (15.8)	38 (34.2)	*p* = 0.039
Outcome at discharge	CKD stage 5, *n* (%)	0 (0)	3 (2.7)	*p* = 0.57
	Death, *n* (%)	0 (0)	3 (2.7)	*p* = 0.57

^a^*n* = 31 with documented weights after 48 h; ^b^*n* = 94 with documented weights after 48 h; ^c^*n* = 21 with dialysis; ^d^*n* = 58 survivors where dialysis was terminated at discharge

*CKD*, chronic kidney disease; *CNS*, central nervous system; *HC*, historic controls; *LDH*, lactate dehydrogenase; *VE*, volume expansion

**Fig. 1:**
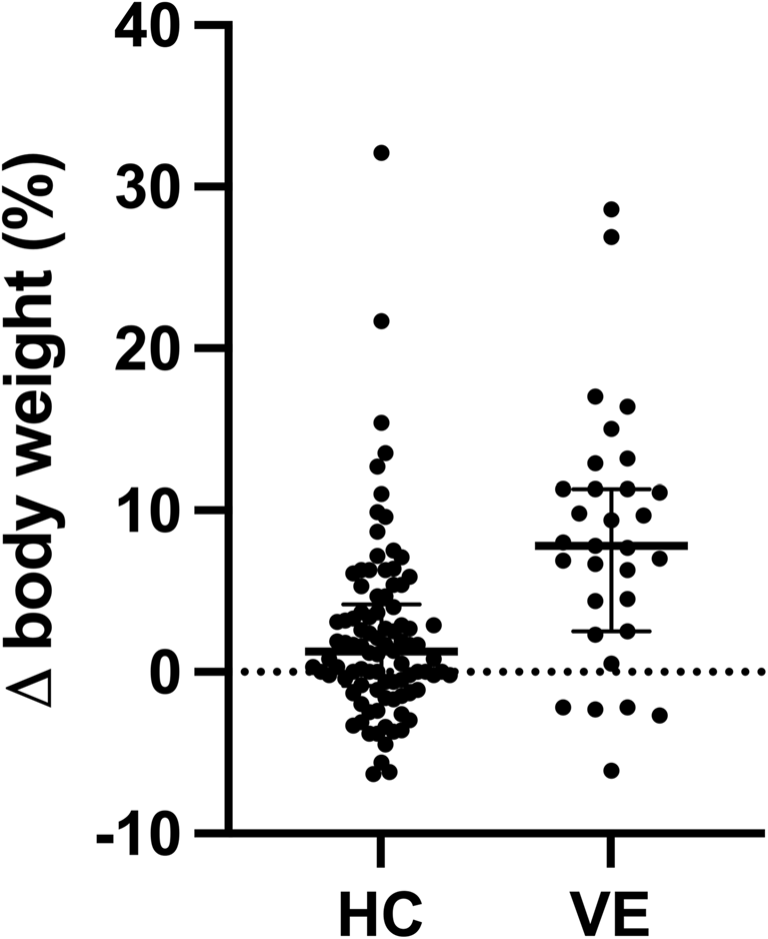
Development of body weight after 48 h in historic controls (HC) and patients treated with volume expansion (VE). Individual values and median are shown

HUS parameters during the course of the disease did not differ between the VE and the HC groups (Table 1). The VE group showed significantly increased relative weight gain over the HC group 48 h after admission (Table 1) (Fig. 1). Routine echocardiography did show signs of fluid overload in 2/26 patients examined within 5 days after admission. There was no difference in dialysis treatment and duration between both groups (Table 1). However, CNS involvement was less frequent in the VE group (Table 1).

### Outcome after the acute phase VE vs. HC

Although this was not statistically significant, deaths or CKD stage 5 at discharge did not occur in the VE group (Table 1). For the combined endpoint CKD stage 5 or death (VE 0/38 (0%) vs. HC 6/111 (5.4%), *p* = 0.34), the reduction in the VE group was also not statistically significant.

### Long-term outcome VE vs. HC

In the VE group 7/38 (18%) and in the HC group 10/108, survivors (9%) were lost to follow-up (*p* = 0.13). Overall follow-up was 2.9 (0.7–4.6) years (VE 1.7 (0.6–3.0) vs. HC 3.3 (1.0–5.1) years, *p* = 0.001). All 3/98 (3%) patients with CKD stage 5 in the HC group received kidney transplantation. Median eGFR in the VE group (*n* = 31) was 151 (123–174) mL/min/1.73 m^2^ vs. 141 (124–167) mL/min/1.73 m^2^ in the HC group (*n* = 95, transplanted patients excluded) (*p* = 0.67). CKD stages 2–5 were present in 4/31 (13%) in the VE group (two patients with CKD stage 2 and two patients with CKD stage 3) vs. 7/98 (7%) in the HC group (*p* = 0.32).

## Discussion

This study shows that STEC-HUS might be mitigated by volume expansion especially regarding CNS involvement. The most severe outcomes of CKD stage 5 and death were not observed in the group treated with volume expansion. The number of cases per year was stable between the HC and the VE groups. The median age of the HC group was significantly higher (Table 1). This was due to the fact that this group includes patients from the O104 outbreak in 2011, where pediatric patients were older [21]. Besides hemoglobin on admission, both groups were comparable regarding their minimum/maximum values on admission and during the course of the disease (Table 1). The dialysis frequency was comparable (Table 1). The higher hemoglobin, also caused by O104 cases, could indicate that patients in the HC group could have been more hemoconcentrated and thus more affected by HUS. Additionally, this could be partially explained by the higher normal hemoglobin values in the age group the O104 cases belonged to.

The median relative weight gain of 7.8% after 48 h in the VE group was between the weight gain described by Ardissino et al. after 48 h (+ 12.5%) and Bonany et al. after 24 h (+ 3.4%) (Table 2) [19, 20]. We did observe an overall frequency of dialysis that was comparable to the studies of Ardissino et al. and Bonany et al. in their control groups [19, 20]. They showed that VE reduced the need for dialysis significantly (Table 2). However, the dialysis frequency and duration were not reduced by VE in our cohort (Table 1). Compared to the studies by Ardissino et al. and Bonany et al., our patients had higher serum creatinine and lower platelets on admission, which might indicate that HUS was already more advanced and thus partially explain the lack of effect of VE on the dialysis rate in our cohort.

**Table 2:** Summary of previous studies regarding volume expansion in STEC-HUS

		Volume expansion	Controls	VE vs. controls
Ardissino et al. [19]	*n*	38	38	
	Treatment	i.v. fluid to reach working weight + 10%	Fluid restriction	
	Median Δ in body weight at 48 h, %	+ 12.5	+ 0	n.a
	Dialysis, %	26.3	57.9	*p* = 0.01
	CNS involvement^a^, %	7.9	23.7	*p* = 0.06
	Death, %	0	5.2	*p* = 0.49
Bonany et al. [20]	*n*	16	19	
	Treatment	i.v. fluid 10–30 mL/kg body weight over 3 h	Fluid restriction	
	Median Δ in body weight at 24 h, %	+ 3.4	+ 1.3	n.a
	Dialysis, %	12.5	47.4	*p* = 0.035
	CNS involvement^b^, %	6.2	15.8	*p* = 0.08
	Death, %	0	5.3	*p* = 1.0

^a^Seizure, coma, (extra-)pyramidal syndromes, confusion; ^b^Seizure. *CNS*, central nervous system; *VE*, volume expansion

In contrast, VE was associated with the reduction of CNS involvement in our cohort. Even Ardissino et al. and Bonany et al. observed a reduction of CNS involvement (Table 2). However, this was not statistically significant in their cohorts [19, 20]. In the article by Bonany et al., this could also be because CNS involvement was defined relatively narrowly by the occurrence of seizures.

As in the previously named studies, there were no deaths observed in the VE group. Additionally, we observed that no patient suffered from CKD stage 5 at discharge in the VE group in our cohort. Probably due to the small number of events, these results were, as in the other studies, not significant. Since mortality in HUS is closely associated with CNS involvement [3], it is plausible that reduction of CNS involvement will lead to reduced mortality as well.

One of the main concerns in VE is potential fluid overload. As the weight gain indicates, we observed edema regularly in patients treated with VE. However, fluid overload with cardiovascular complications was not observed. Ardissino et al. monitored their patients with protocol echocardiography and Bonany et al. by chest x-ray for cardiac size, and neither found signs of fluid overload [19, 20]. We did not observe fluid overload in routine echocardiography in our patients. However, if VE is applied, early transfer to a tertiary center should be considered to allow early planning of dialysis in case of fluid overload.

Previous studies reported variable long-term outcomes of kidney function after STEC-HUS. Recently, Alconcher et al. published a study including 281 patients followed for > 10 years after STEC-HUS [6]. They found that 10% of the patients had CKD stages 2–4 and 4% CKD stage 5 [6]. Even higher rates of CKD stages 2–5 over 20% have been reported [3]. Even though none of the patients with VE died or suffered from CKD stage 5, VE did not influence the long-term outcome regarding CKD stages 2–5 compared to the HC group. However, CKD stages 2–5 were a relatively rare outcome in our cohort compared to previous publications. Ardissino et al. reported even lower numbers for CKD stages 2–5 for their controls (controls 1/38 (2.6%) compared to VE group 0/38 (0%) (*p* = 0.049)) [19]. Follow-up in our VE group was significantly shorter than in the HC group due to the different treatment periods. Therefore, these patients potentially could develop CKD in the future.

Our study has some limitations. First, data were gathered retrospectively and the treatment was not randomized. Due to the retrospective nature of the study, it could not be evaluated in detail, if and why (e.g., ongoing fluid losses) volume expansion in some patients failed. Additionally the small sample size and the inclusion of patients from an outbreak could lead to a potential bias. Cardiovascular monitoring by echocardiography was not conducted in all patients at defined time points. Finally, the sample size was too low to prove that VE statistically significantly reduces the risk of rare events like death or CKD stage 5 due to STEC-HUS.

In conclusion, this study shows that volume expansion may mitigate STEC-HUS regarding CNS involvement. It adds to the body of evidence that adverse outcomes like death and CKD stage 5 might be reduced with volume expansion. Standardized protocols for volume expansion in children with STEC-HUS should be evaluated in prospective trials.

### Supplementary Information

Below is the link to the electronic supplementary material.

Graphical abstract (PPTX 122 KB)

## Data Availability

Datasets are not available because of legal restrictions due to data protection. A request for access can be send to the corresponding author.

## Abbreviations

AKI: Acute kidney injury
CKD: Chronic kidney disease
CNS: Central nervous system
eGFR: Estimated glomerular filtration rate
Hb: Hemoglobin
HC: Historic controls
HUS: Hemolytic uremic syndrome
IQR: Interquartile range
LDH: Lactate dehydrogenase
KRT: Kidney replacement therapy
STEC: Shiga toxin-producing *Escherichia coli*
Stx: Shiga toxin
VE: Volume expansion
