# Supplementary material for: Volume expansion mitigates Shiga toxin-producing E. coli-hemolytic uremic syndrome in children
Source: Pediatr Nephrol. 2024 Jan 19;39(6):1901–7. doi: 10.1007/s00467-023-06276-3 (PMC11026235; doi:10.1007/s00467-023-06276-3)
Supplement: Supplementary file 1 — Graphical abstract (PPTX 122 KB) [file 467_2023_6276_MOESM1_ESM.pptx]

## Slide 1
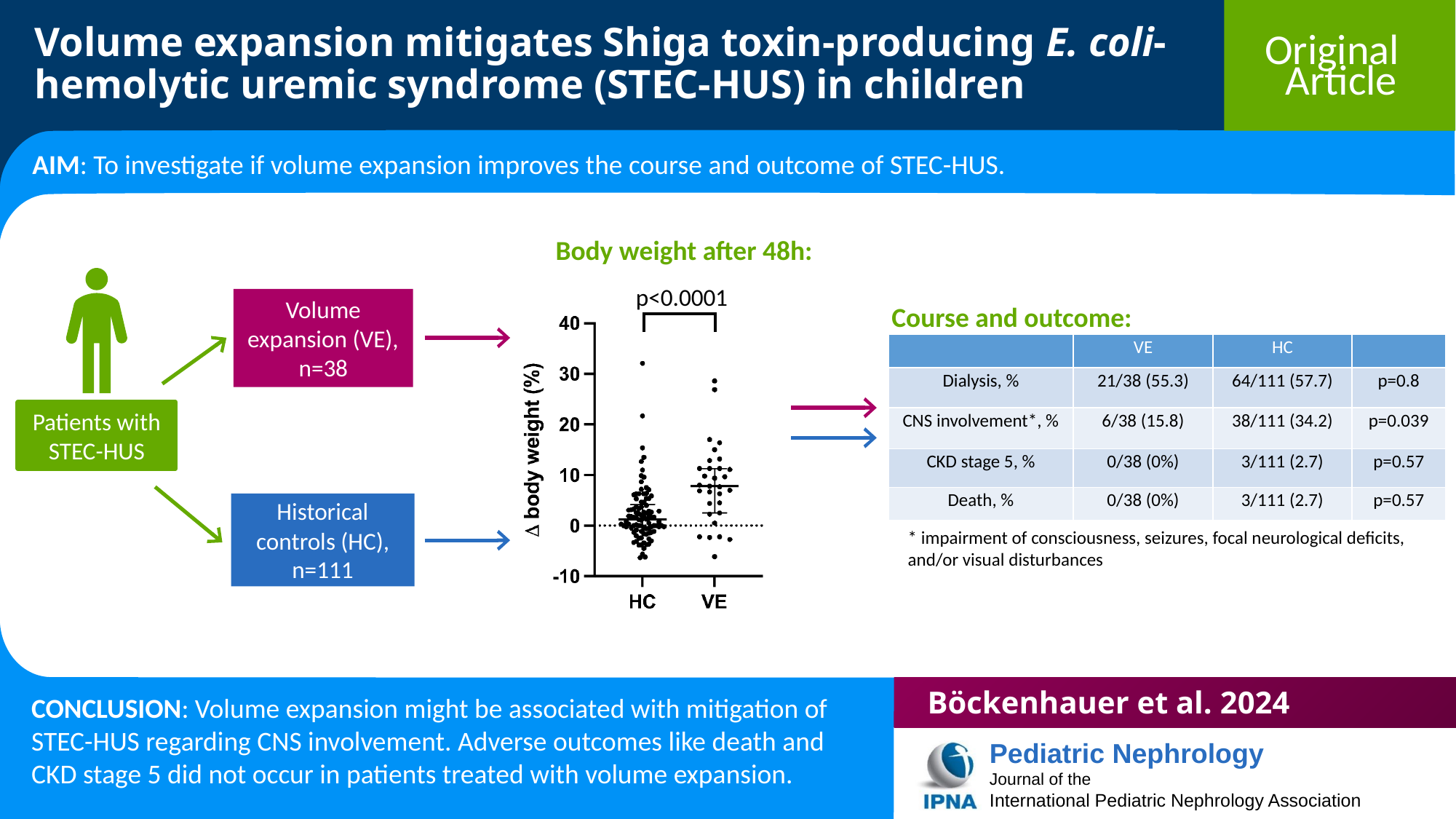

Volume expansion mitigates Shiga toxin-producing E. coli-
hemolytic uremic syndrome (STEC-HUS) in children
AIM: To investigate if volume expansion improves the course and outcome of STEC-HUS.
Body weight after 48h:
p<0.0001
Volume expansion (VE),
n=38
Course and outcome:
| | VE | HC | |
| --- | --- | --- | --- |
| Dialysis, % | 21/38 (55.3) | 64/111 (57.7) | p=0.8 |
| CNS involvement\*, % | 6/38 (15.8) | 38/111 (34.2) | p=0.039 |
| CKD stage 5, % | 0/38 (0%) | 3/111 (2.7) | p=0.57 |
| Death, % | 0/38 (0%) | 3/111 (2.7) | p=0.57 |
Patients with STEC-HUS
Historical controls (HC), n=111
* impairment of consciousness, seizures, focal neurological deficits, and/or visual disturbances
Böckenhauer et al. 2024
CONCLUSION: Volume expansion might be associated with mitigation of STEC-HUS regarding CNS involvement. Adverse outcomes like death and CKD stage 5 did not occur in patients treated with volume expansion.
